# Dynamic 3D meta-holography in visible range with large frame number and high frame rate

**DOI:** 10.1126/sciadv.aba8595

**Published:** 2020-07-10

**Authors:** Hui Gao, Yuxi Wang, Xuhao Fan, Binzhang Jiao, Tingan Li, Chenglin Shang, Cheng Zeng, Leimin Deng, Wei Xiong, Jinsong Xia, Minghui Hong

**Affiliations:** 1 Wuhan National Laboratory for Optoelectronics, Huazhong University of Science and Technology,1037 Luoyu Road, Wuhan 430074, China.; 2 Department of Electrical and Computer Engineering, National University of Singapore, Engineering Drive 3, Singapore 117576, Singapore.

## Abstract

The hologram is an ideal method for displaying three-dimensional images visible to the naked eye. Metasurfaces consisting of subwavelength structures show great potential in light field manipulation, which is useful for overcoming the drawbacks of common computer-generated holography. However, there are long-existing challenges to achieving dynamic meta-holography in the visible range, such as low frame rate and low frame number. In this work, we demonstrate a design of meta-holography that can achieve 2^28^ different holographic frames and an extremely high frame rate (9523 frames per second) in the visible range. The design is based on a space channel metasurface and a high-speed dynamic structured laser beam modulation module. The space channel consists of silicon nitride nanopillars with a high modulation efficiency. This method can satisfy the needs of a holographic display and be useful in other applications, such as laser fabrication, optical storage, optics communications, and information processing.

## Teaser

A space channel design approach achieves dynamic meta-holography with high frame rate and large frame number.

## INTRODUCTION

As a technology that records and reconstructs wavefronts of light, holography is an ideal approach for naked-eye three-dimensional (3D) display (*1*), optical data storage (*2*), and optical information processing (*3*). However, the traditional hologram cannot create a holographic reconstruction of a virtual object or dynamic display. To overcome these limitations, in 1966, Brown and Lohman (*4*) invented computer-generated holography (CGH), which uses physical optics theories to calculate the phase map on the interference pattern. Furthermore, by using digital devices, such as a spatial light modulator (SLM) or a digital micro-mirror device (DMD), CGH can also perform dynamic holographic display (*5*, *6*). However, there are long-existing challenges for CGH with SLM/DMD for large pixel-size applications, such as small field of view (FOV), twin imaging, and multiple orders of diffraction (*7*, *8*).

Recently, with the enormous development of nanofabrication technology, metamaterials and metasurfaces have ushered in a new era for hologram study and other research fields in engineering optics 2.0 (*9*). Metamaterials consist of subwavelength artificial structures that perform innovative functions, exceeding the limitations of bulk materials. The fabrication of 3D metamaterials is extremely difficult; thus, metasurfaces play a notable role as optical devices in the visible range. As a type of 2D metamaterial consisting of subwavelength nanostructures, metasurfaces offer a powerful tool to achieve light modulation of amplitude, phase, and polarization. It is shown that conventional optical rules should be recast into a more general form (*10*). Research on metasurfaces can be categorized into static and dynamic metasurfaces. The design of dynamic or active metasurfaces is based on using different materials and mechanisms, such as phase-change materials (*11*), liquid crystals (*12*), light-induced (*13*), mechanical strain (*14*), charge injection (*15*), thermo-optic effect (*16*), chemical and structural approaches (*17*, *18*), and so on. Today, metasurfaces have been used to fabricate many different types of functional devices, such as metalens (*19*, *20*), beam splitters (*21*), catenary optical elements (*22*), and orbital angular momentum (OAM) devices (*23*).

There are several major advantages to metasurface holography in terms of its subwavelength unit structures, including a large FOV, high resolution, and the elimination of high orders of diffraction (*7*, *24*). Meta-holography can be categorized into three categories representing different physical mechanisms, namely, phase-only meta-holograms (*25*), amplitude-only holograms (*26*), and complex-amplitude holograms (*27*). Most meta-holography studies in the visible range are designed as static devices, which can only show a single frame with one piece of metasurface. However, dynamic design is essential for ideal meta-holographic smooth display. To achieve this goal, there are two important considerations. The first is the frame number, which refers to the number of different frames that a single meta-hologram element can show. The second is the frame rate (reciprocal of switch time between two frames) of the meta-holographic display, which can be quantified by the number of “frames per second (fps).” The final goal is that discrete reconstructed holographic frames can be perceived and interpreted as a smooth video, owing to eye persistence. It is generally accepted that video displays of frame rates higher than 24 fps are continuous to the human eye (*28*). A higher frame rate corresponds to a finer and smoother video display.

Table 1 provides an overview of the state-of-the-art development of dynamic meta-holography in the visible range. Dynamic meta-holography can be categorized into two groups with different operating principles. The first group uses active metasurfaces, which would undergo changes in their physicochemical properties via external controls. Many different methods have been developed to realize this goal, such as use of phase-change materials [e.g., Ge_2_Sb_2_Te_5_ (GST)] (*11*, *29*), application of stretchable substrates (*14*), modification of optical characteristics through chemical reactions (*17*), and rewriting graphene oxide metasurfaces with femtosecond lasers (*13*). The other group is using static multiplexing metasurface. There are various multiplexing methods that have been applied in recent research, such as wavelength (*30*, *31*), incident angle (*32*), and polarization multiplexing (*33*–*36*). The complex modulation of incident light has been used as a multiplexing factor in instruments, such as OAM devices (*37*). In addition to the aforementioned meta-holography with a single kind of multiplexing method, a multiple multiplexing method showing recording of 63 holographic images within the same hologram multiplexed by means of wavelength and polarization is also proposed (*38*). All these representations of dynamic meta-holography are progressive and inspiring. However, there are still critical challenges to realizing dynamic meta-holography in the visible range, as shown in Table 1. First, as discussed above, it is essential to display many different frames with one piece of a meta-hologram element to achieve an authentically appearing holographic video; however, most current designs can only display a few different frames experimentally. Second, the modulation time of most endeavors are too long to show smooth holographic videos. Table 1 demonstrates that in most holographic studies, the frame rate of dynamic meta-holography is never mentioned. Recent studies regarding OAM multiplexing meta-holography show that the achievement of a relatively smooth dynamic hologram is expected with 2^10^ frames and 60 fps (*37*), and the reconstructed image consists of a solid-spot array. This design is very creative and more suitable for all-optical encryption or storage, rather than a holographic display. Ultimately, there is still a lack of high-efficiency dynamic meta-holography and excellent display quality in the visible range, capable of exhibiting smooth holographic videos with a large frame number and high frame rate.

**Table 1:** Summary of different dynamic meta-holography. “/” means no related data in the references.

**Operation principle**	**Methods**	**References**	**Working mode**	**Working** **wavelength (nm)**	**Frames number**	**Frame rate (fps)**
**Active metasurfaces**	Phase-change material	(*11*)	Reflection	473, 532, and 660	No limitation in theory	/
	Stretchable substrate	(*14*)	Transmission	633	6	/
	Chemical reaction	(*17*)	Reflection	633	10	1/40
	Rewriting metasurface	(*13*)	Transmission	405, 532, and 632	No limitation in theory	/
**Multiplexed** **metasurfaces**	Wavelength	(*30*)	Transmission	380–780	7	/
		(*31*)	Transmission	473, 532, and 633	3	/
	Angle	(*32*)	Transmission	405	8	/
	Polarization	(*33*)	Reflection	405, 633, and 780	3	/
		(*34*)	Transmission	800	7	/
		(*35*)	Transmission	532	2	/
		(*36*)	Reflection	475–1100	5	/
	OAM	(*37*)	Transmission	633	2*^N^* (for *N*-bit OAM-multiplexing hologram device)	60
	Space channel	Current work in this paper	Transmission	633	2*^N^* (for *N*-bit space channel multiplexing hologram device)	9523

In this study, we demonstrate a new design of meta-holography in the visible range based on a space channel multiplexing metasurface that can achieve 2^28^ different holographic frames and a very high frame rate (maximum frame rate, 9523 fps). Furthermore, a high modulation efficiency (greater than 70%) for each space channel has been achieved through the application of silicon nitride (SiN*_x_*) nanopillar building blocks for the construction of the metasurface.

## RESULTS

### Design and realization of dynamic SCMH

The inspiration for the design of the space channel meta-hologram (SCMH) comes from the comparison between the dynamic meta-hologram and common 2D display technologies. The ideal means to achieve dynamic meta-holography is to perfectly control each nanostructure of the metasurface. This means that each pixel of the element needs to be controlled independently at high speed, just as how light-emitting diode or liquid crystal display screens function. Recently published works demonstrate metasurfaces with individually controlled linear pixels, showing dynamic beam steering and focusing capabilities (*39*, *40*), which suggest a feasible path to achieve dynamic holography in the future. Besides these pixel display screens, there are two other methods used to achieve dynamic 2D display. One is to divide the entire graph into many different subgraphs and combine them at different times, e.g., the digital tube display on an electronic scoreboard or an electronic meter. The other is to display different frames from a continuous video at different times, e.g., conventional movies recorded and projected as cinefilms. It can be concluded that both are space channel methods.

**Fig. 1:**
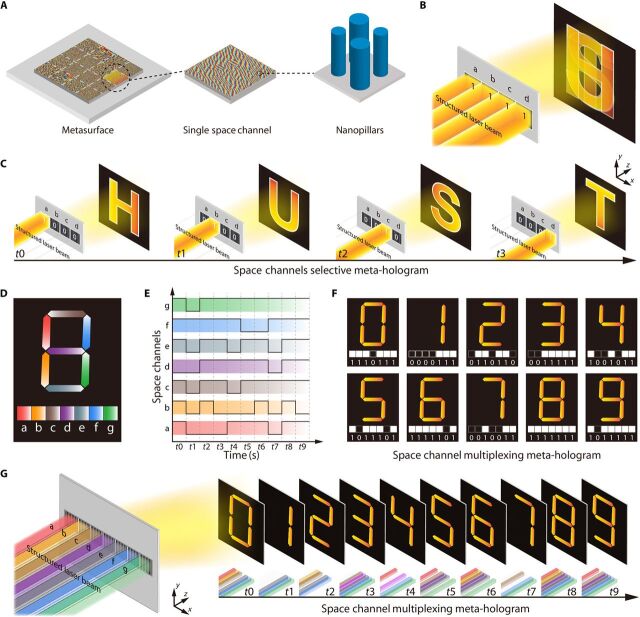
Principle of dynamic space channel meta-hologram. (**A**) Structure of space channel meta-hologram element. (**B** and **C**) Space channel selective meta-hologram design. All reconstructed images overlap each other if all space channels were opened at the same time (B). Dynamic meta-holographic display can be achieved by opening space channels in the designed sequence (C). (**D** to **G**) Space channel multiplexing meta-hologram design. The reconstructed images of different space channels are subgraphs of a whole graph (D). Different space channels are opened in different time sequences to form different space channel combinations (E), which reconstruct different images (F) to achieve dynamic meta-holographic display (G).

The physical mechanism of the SCMH is demonstrated in note S1 and illustrated in Fig. 1. The traditional meta-hologram design involves the calculation of the corresponding phase map or amplitude map of the reconstructed target objects with a mathematical algorithm (e.g., Gerchberg-Saxton algorithm) over the entire fabricated metasurface region. To create an SCMH, the metasurface is divided into *N* different space channels, which consist of thousands or millions of nanopillars (Fig. 1A). There are two different types of SCMH designs. The first one is a space channel selective meta-hologram. In this design, if different space channels were simultaneously opened, all the reconstructed images of the different space channels would overlap each other (Fig. 1B). By controlling the structured laser beam to open different space channels in a designed sequence, continuous frames of the holographic video are displayed at the designated time (Fig. 1C). The other design is the space channel multiplexing meta-hologram, in which the reconstructed target images of the different space channels are subgraphs of the entire holographic graph (Fig. 1D). Different space channels are opened at different times according to a predetermined sequence; thus, there are 2*^N^* combinations for *N*-bit space channels at the same time (Fig. 1E). Different space channel combinations reconstruct different holographic images (Fig. 1G). By changing the structured laser beam to open different space channel combinations at different times at high speed, a dynamic meta-holographic display can be shown in a smooth and fine manner (Fig. 1G).

**Fig. 2:**
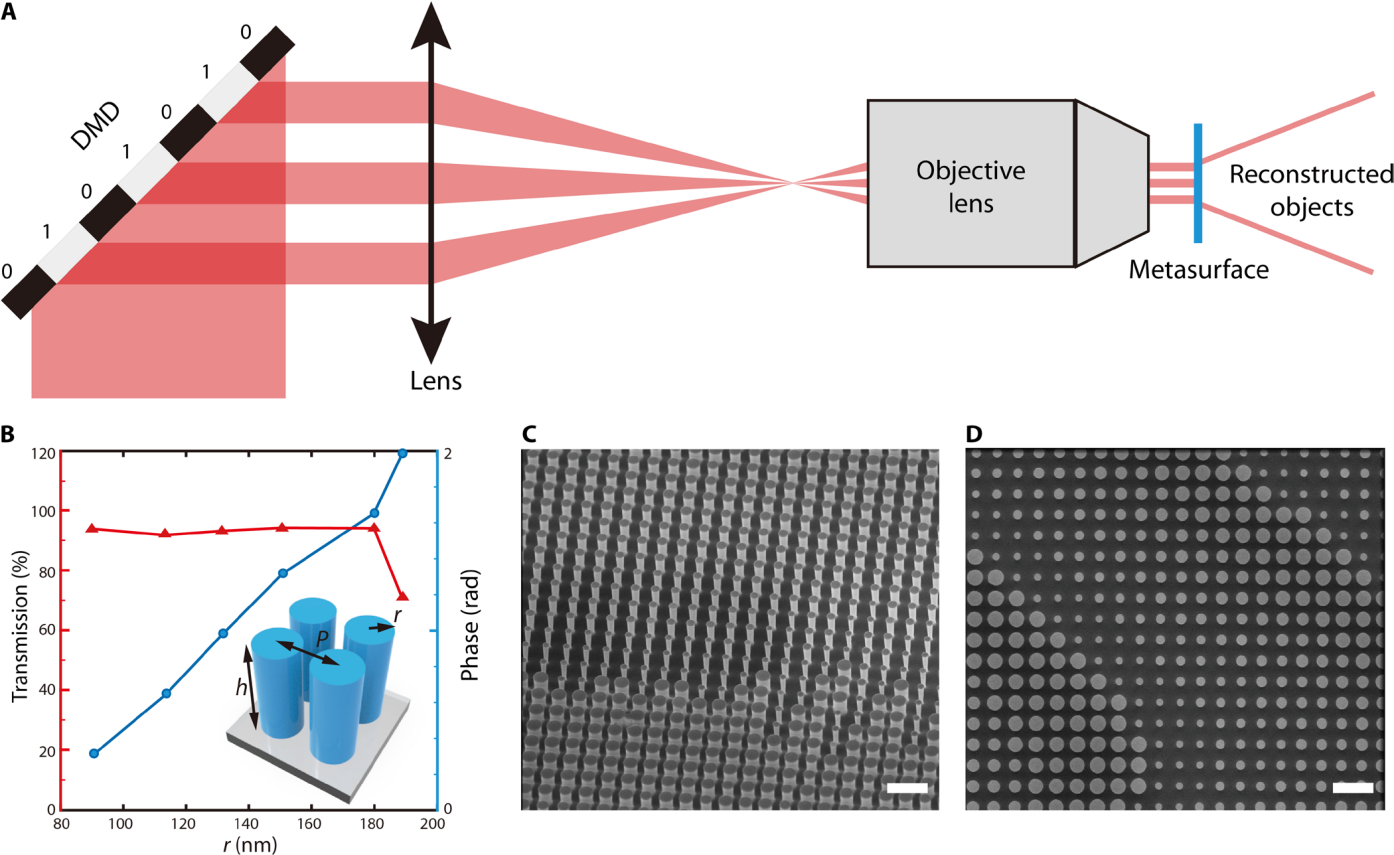
Realization of dynamic SCMH. (**A**) Dynamic space beam coding module. DMD modulates the incident light at a high speed, e.g., maximum 9523 Hz in our experiment. The lens and microscope objective perform as a 4f system to narrow the coded incident beam to illuminate the different regions of the metasurface. (**B**) Geometrical diagram of SiN*_x_* nanopillars and characterization of amplitude transmission efficiency and phase response of SiN*_x_* nanopillars as functions of nanopillar radius at a wavelength of 633 nm. The illustration is a geometrical diagram of SiN*_x_* nanopillars. (**C** and **D**) Scanning electron microscopy (SEM) images of the fabricated results. Scale bars, 1 μm.

The above discussion demonstrates that there are two important aspects to achieving our dynamic meta-holography design. The first is the dynamic beam modulation used to code the space distribution of the incident structured laser beam. The module can be achieved by a projection system composed of a DMD, a lens, and a microscope objective, as shown in Fig. 2A. The incident light is modulated by the DMD at high speed, e.g., a maximum of 9523 Hz in our experiment. The lens and microscope objective perform as a 4f system to narrow the structured incident beam to open the different space channels of the metasurface. Another essential aspect is the static space channel multiplexing metasurface with high efficiency in the visible range. In this study, the static metasurface consists of SiN*_x_* nanopillars, as shown in Fig. 2B. The absorption coefficient of the SiN*_x_* material is sufficiently small, such that SiN*_x_* is nearly transparent in the visible range. Its refractive index *n* is near 2, which is far greater than that of the normal glass materials. These characteristics make SiN*_x_* material suitable for the design of a high-efficiency metasurface with an equivalent refractive index in the visible range. The heights of SiN*_x_* nanopillars are all the same at 700 nm, and the periods of the rectangular lattice are 500 nm, whereas the radii vary from 90 to 188 nm. The nanopillars were simulated by the finite-difference time-domain (FDTD) method, and six proper radii were chosen for the fabrication. The characterization of the amplitude transmission efficiency and phase response of the SiN*_x_* nanopillars are shown in Fig. 2B as functions of nanopillar radius at a wavelength of 633 nm. Figure 2B indicates that the transmission efficiency almost maintains a high constant value above 90% for the selected radii, but it falls down at the maximum radius of 188 nm, whereas the phase response varies from 0 to 2π. Figure 2 (C and D) presents the scanning electron microscopy (SEM) images of the fabricated results. The scale bar in the SEM images is 1 μm.

### Dynamic space channel multiplexing meta-hologram

**Fig. 3:**
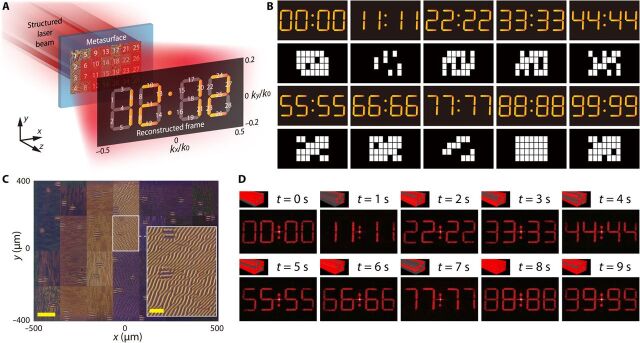
Design and experimental results of 28-bit dynamic space channel multiplexing meta-hologram. (**A**) Structured laser beam opens specific space channel combinations and reconstructs the target image. (**B**) First and third rows: 10 typical examples varying from 00:00 to 99:99; second and fourth rows: corresponding space channel coding pattern of DMD. (**C**) Optical image of fabricated metasurface and enlarged view of one space channel. Scale bars, 100 and 30 μm. (**D**) Experimental results of dynamic space channel multiplexing meta-hologram and corresponding pattern of structured laser beam.

As discussed above, one of the designs for dynamic display involves dividing the entire picture into subgraphs and illustrating different frames by a combination of different subgraphs. This method can also be used in the design of the space channel multiplexing meta-hologram. In this study, a metasurface holographic digital tube display system is designed and demonstrated, as shown in Fig. 3A. The entire reconstructed target image is the digital tube pattern of “88:88,” which consists of 28 subgraphs. Accordingly, the metasurface is divided into 28 different space channels, which reconstruct the corresponding subgraphs as marked by numbers (see fig. S1 for detailed design). The example of frame “12:12” is demonstrated. By coding the space distribution of the incident structured laser beam, the metasurface can reconstruct a tremendous number of different frames, representing a type of shared-aperture design. This is a 28-bit design, bringing the total frame number to 2^28^ = 268,435,456.

Ten typical examples are presented in Fig. 3B to demonstrate the design. The illustrations of the reconstructed target images vary from 00:00 to 99:99 in the first and third rows. Meanwhile, the second and fourth rows exhibit the corresponding space channel coding pattern of the DMD. Notably, there are dark gaps between the coding patterns of the DMD, as shown in the illustrations, whereas there is no gap between the subregions of the fabricated metasurface. There is a little divergence in the narrowed laser beams that illuminate the subregions, owing to geometrical optical aberration and diffraction. Meanwhile, dark gaps could restrain the cross-talk of the adjacent subregions. The size of each space channel is 150 μm by 200 μm, and the entire size of the fabricated metasurface region is 1050 μm by 800 μm (Fig. 3C). The frame rate of the meta-holographic digital tube display depends on the switch time of the DMD coding pattern. In our experiment, the minimum DMD switch time is 105 μs; thus, the frame rate could vary from 0 to 9523 fps, which is much higher than that associated with the limitation of vision persistence. The experimental results are presented in Fig. 3D, and the frame rate in this experiment is 1 fps. It is demonstrated that our design could achieve a smoothly dynamic meta-holographic display (see movie S1).

### Dynamic space channel selective meta-hologram

**Fig. 4:**
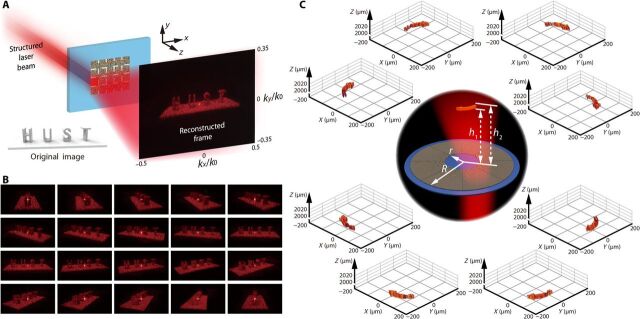
Design and experimental results of space channel selective meta-hologram. (**A**) Structured laser beam opens a specific space channel in the designed sequence, and (**B**) continuous frames of a holographic video are displayed. (**C**) A dynamic 3D holographic display is achieved by a space channel selective meta-hologram.

Another design in this study is that of the dynamic space channel selective meta-hologram, which is similar to conventional movies recorded and projected as cinefilms. The metasurface sample is divided into many space channels, which would represent the reconstruction of different frames from a continuous video. In this design, 20 continuous frames from a short video, showing the rotation of four capital letters “HUST” (see fig. S2 for detailed design), are selected as the reconstructed frames of a dynamic meta-hologram, as shown in Fig. 4A (see fig. S3 for detailed phase map). The incident structured laser beam is modulated by a DMD as a space-scanning beam and illuminates different single space channels of the metasurface in the designed sequence. Then, the reconstructed frames change with time to display the dynamic meta-holographic movie, whereas the frame rate of the holographic video depends on the switch time of the DMD. The experimental results for each frame are presented in Fig. 4B. The short meta-holographic video demonstrates the practicability of this method (see movie S2).

The dynamic space channel selective meta-hologram design can be used to display 2D and 3D holographic videos. A metasurface is designed for the 3D holographic video display, as shown in Fig. 4C. The entire annular meta-holographic element is divided into eight space channels, and each space channel is designed to reconstruct a 3D arrow in free space. The geometrical parameters are marked in Fig. 4C, and the internal radius of the annular metasurface is *r* = 150 μm, whereas the outer radius *R* = 450 μm. The reconstructed 3D arrows are designed in the central circle with radius 125 μm and a height between *h*_1_ = 2000 μm and *h*_2_ = 2020 μm. Eight 3D arrows are positioned end to end in free space. The reconstructed light field of each 3D arrow is detected by a homemade microscope along the *z* axis (see fig. S4 for detailed phase map and experimental results). This demonstrates that this design can be used in smooth meta-holographic display (see movie S3).

## DISCUSSION

Dynamic meta-holography with a large frame number and high frame rate in the visible range has been achieved on the basis of the dynamic space coding of the incident beam by the DMD and a space division multiplexing metasurface design. Two different designs are demonstrated in this paper, namely, dynamic space channel multiplexing and selective meta-hologram. The dynamic space channel multiplexing meta-hologram could display 2*^N^* different frames (more than 200 million in this study). The dynamic space channel selective meta-hologram could show complex 2D and 3D holographic videos in the visible range. All three of these designs can achieve a high frame rate (0 to 9523 fps), which is far beyond the limitation of visual residue, such that our method can display ultrafine and smooth holographic videos. The metasurface consists of SiN*_x_* nanopillars that are designed by the equivalent refractive index and simulated by FDTD. Notably, each space channel of the as-fabricated metasurfaces features a high efficiency (greater than 70%) in the visible range (see note S2 and fig. S5 for detailed calculation). Each space channel can be opened partly by modulating the spatial duty ratio (see fig. S6 for related signal-to-noise ratio analysis). The number of space channels *N* depends on the fabrication size, FOV of the objective, and minimum size of each space channel. Creating thousands of space channels is practicable with our design method (see note S3 for a detailed analysis). A large frame number, high frame rate, and high efficiency not only endow this metasurface method with the capability to satisfy the demanding requirements of complex and smooth holographic display in the visible range but also provide promise in many applications, including laser fabrication, optical storage, optics communications, and information processing.

## MATERIALS AND METHODS

### Fabrication of SiN*_x_* metasurface

The fabrication of the SiN*_x_* metasurface starts from a glass wafer substrate with a thickness of 500 μm (fig. S7). A silicon nitride layer (*n* = 2.023 at 633 nm) of 700-nm thickness is deposited by plasma-enhanced chemical vapor deposition onto the substrate. Then, a chromium layer of 20 nm is deposited by electron beam evaporation on top of the SiN*_x_* layer as a hard mask. Next, a 200-nm photoresist layer (CSAR62) is spin-coated onto the top of the Cr layer. The hologram pattern is written by electron beam lithography (Vistec: EBPG 5000 Plus) and implemented into the photoresist layer after development. The pattern is then transferred into the Cr hard mask layer by inductively coupled plasma (ICP) etching (Oxford Plasmalab: System 100-ICP-180), and the residual photoresist is stripped off by an oxygen plasma stripper (Diener electronic: PICO plasma stripper). Last, the pattern is transferred into the SiN*_x_* layer by the next ICP process, and the remaining Cr is removed by Cr corrosion solution. The Cr layer is used as a hard mask because of the extremely high etching selectivity between Cr and SiN*_x_*.

### Optical setup

The optical components and setup of the dynamic space division multiplexing metasurface are shown in fig. S8. The He-Ne laser (Pacific Lasertec, 25-LHP-991-230) at the wavelength of 633 nm propagates through a spatial pinhole filter and collimating lens and becomes an expanded laser beam with suitable beam quality. Then, the expanded laser beam is modulated by a DMD (Texas Instruments, DLP6500FYE) at high speed. The coded beam propagates through the 4f system consisting of a lens and a microscope objective. The reconstructed holographic frames are collected by Fourier lens or objective lens and recorded by CCD.

## Supplementary Material

aba8595_Movie_S1.mp4

aba8595_SM.pdf

aba8595_Movie_S2.mp4

aba8595_Movie_S3.mp4

## SUPPLEMENTARY MATERIALS

Supplementary material for this article is available at http://advances.sciencemag.org/cgi/content/full/6/28/eaba8595/DC1
