# Supplementary material for: Dynamic 3D meta-holography in visible range with large frame number and high frame rate
Source: Sci Adv. 2020 Jul 10;6(28):eaba8595. doi: 10.1126/sciadv.aba8595 (PMC7351452; doi:10.1126/sciadv.aba8595)
Supplement: aba8595_SM.pdf [file aba8595_SM.pdf]

Science Advances
AAAS

advances.sciencemag.org/cgi/content/full/6/28/eaba8595/DC1

Supplementary Materials for

Dynamic 3D meta-holography in visible range with large frame number and high frame rate

Hui Gao, Yuxi Wang, Xuhao Fan, Binzhang Jiao, Tingan Li, Chenglin Shang, Cheng Zeng, Leimin Deng, Wei Xiong*, Jinsong Xia*, Minghui Hong

*Corresponding author. Email: weixiong@hust.edu.cn (W.X.); jsxia@hust.edu.cn (J.X.)

Published 10 July 2020, *Sci. Adv.* **6**, eaba8595 (2020)
DOI: 10.1126/sciadv.aba8595

The PDF file includes:

Notes S1 to S5
Figs. S1 to S9
Legends for movies S1 to S3

Other Supplementary Material for this manuscript includes the following:

(available at advances.sciencemag.org/cgi/content/full/6/28/eaba8595/DC1)

Movies S1 to S3

Supplementary Note

Note S1. The physical mechanism of the space-channel meta-hologram (SCMH)

The corresponding target reconstructed object for every independent space-channel is different but associated with each other. Different target reconstructed objects are either subgraphs of a whole holographic graph or continuous frames of a holographic video.

As marked in Fig. 1, n^{th} space-channel can reconstruct the corresponding n^{th} object. By modulating the spatial distribution of incident structured laser beam to open different space-channels at different time, the reconstructed frames would also change with time. Notably, the reconstructed frames can be 2D pictures as well as 3D objects.

$$E(x,y,t)=\left\{\sum_1^N F(t,n)A\left[x(n),y(n)\right]\right\}*\left\{\sum_1^N e^{-i\varphi[x(n),y(n)]}\right\}. \quad (1)$$

Eq. (1) shows the basic idea of dynamic SCMH design. $E(x,y,t)$ means the modulated complex amplitude by designed metasurface at time t. The phase map of static metasurface is

$$\phi(x,y)=\sum_1^N \varphi\left[x(n),y(n)\right], \quad (2)$$

which consists of N different space-channels with different corresponding reconstructed target objects. $A(x,y,t)$ means the equal amplitude of incident planar beam, $F(t)$ is the binary function which can only be equal to 1 or 0. So we have

$$A(x,y,t)=\sum_1^N F(t,n)A\left[x(n),y(n)\right], \quad (3)$$

which means the modulated light distribution of incident structured laser beam at time t. The total number of different frames for N space-channel design is 2^N.

Note S2. Definition of the efficiency of each space-channel

The transmission coefficient of designed nanopillars is more than 92% for all six radii as shown in Fig. 2B. But that is not absolute efficiency of metasurface because of energy loss, such as the reflection and absorption of substrate, undesired scattering of nanostructures. The measured absolute transmission efficiency is 77.8%. What's more, there's a little non-modulated zero-order laser beam generating light spot in the center of display area as shown in Fig. 3D and Fig. 4B. The samples in Fig. 3D and Fig. 4B are designed in Fraunhofer diffraction theory, and the reconstructed images enlarge quickly along z-axis. Meantime, the spot-size of non-modulated laser beam keeps nearly same along z-axis. Thus, the intensity of non-modulated laser beam can be measured by power-meter at distance far away from metasurface (fig. S5). In our experiment, z=0.5 m is far enough to ignore the influence of modulated light. It can be found that the non-modulated laser beam makes up 9.8% of all transmitted light. The total efficiency of each space-channel is 77.8%×(1-9.8%)=70.2%.

To be noticed that a small percentage of diffraction light is the contribution of undesired diffraction orders due to the relatively large period of pillars which is larger than λ/n (λ is light wavelength; n is refractive index of SiN_x). The undesired diffraction order can be eliminated by more advanced fabrication techniques with higher spatial resolution.

Note S3. Description of the number *N* of space-channels

Figure 2 shows the design of structured laser beam generation module. In this design, the number of space-channels *N* is depended on the fabricated size of metasurface, field of

view of objective, and minimum size of each space-channel. In our experiment shown in Fig. 3, the objective field number is 26.5, which means the diameters of field of views are 6.625 mm for 4× and 2.65 mm for 10×. If the size of each channel is 100×100μm^2, the total number of space-channels is more than three thousands for 4×and five hundreds for 10×, respectively.

The DMD is a chip with a surface built of a rectangular array of micromirrors. The size of each micromirror is 7.65×7.65 μm^2. Thus, if the shape of each space-channel is also rectangular, DMD can also be used independently without lens and objective as structured laser beam generator. In this way, the number of space-channels N is only depended on the sizes of fabricated metasurface and single space-channel. The size of fabricated metasurface achieves centimeter-scale is practical nowadays (*Opt. Express* 26, 1573-1585, 2018; *Nano Lett.*, 19, 8673-8682, 2019). So, N being up to ten thousand is also possible.

Note S4. Brief discussion of potential applications of the dynamic meta-hologram

The new approach proposed in the manuscript is not intended to achieve all applications of dynamic meta-holography. It combines the advantages of meta-hologram and DMD, and is quite suitable for many specific applications. Here we take two examples to show its potential applications.

First, holographic display. With no doubt that metasurface with an individual pixel/nanostructure control is the general solution for nearly all meta-holographic applications. But the related research on active metasurfaces is still in infant stage nowadays. And the general solution may not be the best solution for all applications. The

demonstrated approach is suitable for the display scenarios with fixed elements as proposed in this manuscript, such as traffic signal system, warning symbols, indicator system, loop display, time display system, and so on.

Second, micro/nano-scale additive manufacturing including projection lithography, two-photon polymerization, et al. For example, there are plenty of works discussing about applying holographic reconstruction method in micro-scale 3D printing. However, most of the works achieved holographic reconstruction using spatial light modulator (SLM). The frame rate of SLM is too low to satisfy the high-speed fabrication system. By using the approach as proposed in our manuscript, different holographic reconstructed light field from different spatial channels could perform as the basic “building blocks” of 3D printing. And ultra-high frame rate of this approach can achieve high-speed 3D micro/nano-fabrication.

Note S5. Brief discussion about tapering effects of nanopillars

The tapering shape of nanopillars is caused by over-etching in our fabrication process. The sizes of tapered shapes of six different radii are measured from zoomed-in SEM images. And the updated simulation results of phase modulation and transmission of the tapered nanopillars are shown in Fig. S9. Figure S9 (A) shows the amplitude transmission efficiency and phase response of SiN_x nanopillars with no fabrication errors while Fig. S9 (B) presents the results with tapered shaped fabrication errors. There are phase shifts resulting from tapered fabrication errors which would cause background noise as well as non-modulated zero order as shown in Fig. S9 (C).

Interestingly, the transmission efficiency of tapered nanopillars is higher than cylinder nanopillars especially at the maximum radius condition. It is believed that the tapered nanopillars perform as graded-index materials which reduces the reflection.

A brief analysis has been performed to improve the fabrication errors. Due to the micro-loading effect in the etching process, the etching rate of reactive ions is not constant. In the same design pattern, different radii of nanopillars will lead to different etching rates. Therefore, in the region with a smaller radius, the etching rate is faster and leads to more serious over-etching. The micro-loading effect can be reduced to improve tapered shape fabrication errors by optimizing the reaction chamber pressure, the flow rate and ratio of etching gas, and RF power.

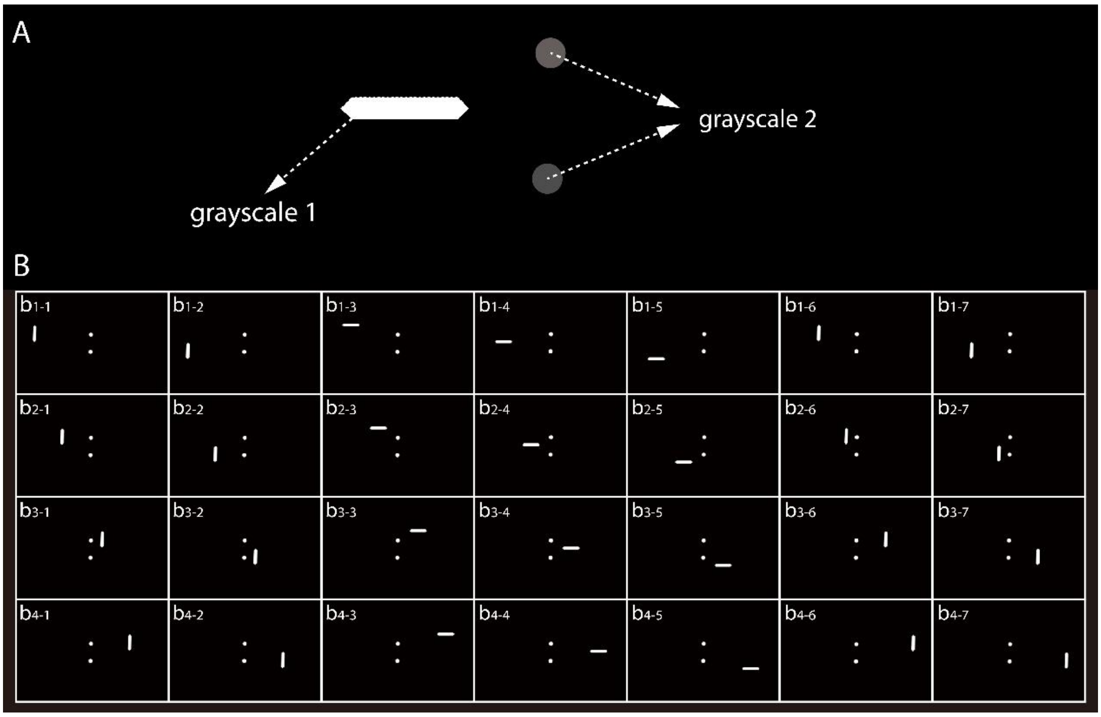

Fig. S1. Designed target images of different space-channels for dynamic space-channel multiplexing meta-hologram sample. (A) Each target pattern of space-channel consists of one tube pattern and two circular dots with different grayscales. The tube patterns are different from each other in different sub-regions as marked in Fig. 3 while two circular dots are all the same. The intensity changes of two reconstructed circular dots can be observed from Fig. 3D (e. g. difference between "00:00" and "11:11"), which demonstrates that the intensity of reconstructed images depends on the superposition of different space-channels as well as designed grayscales. **(B)** Designed target patterns for different space-channels.

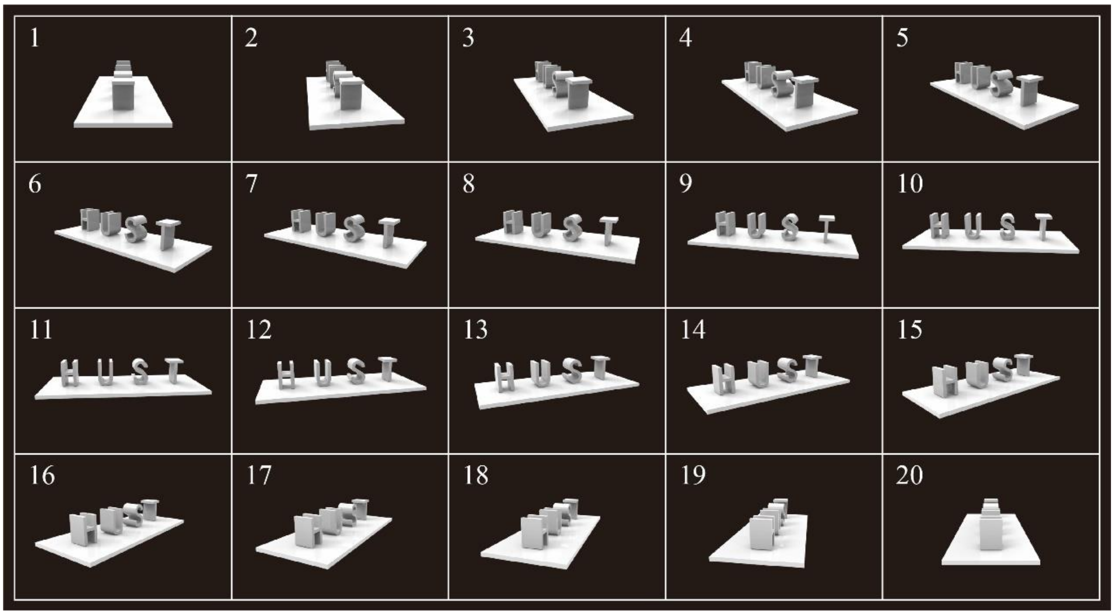

Fig. S2. The designed target patterns of different space-channels for dynamic space-channel selective meta-hologram sample.

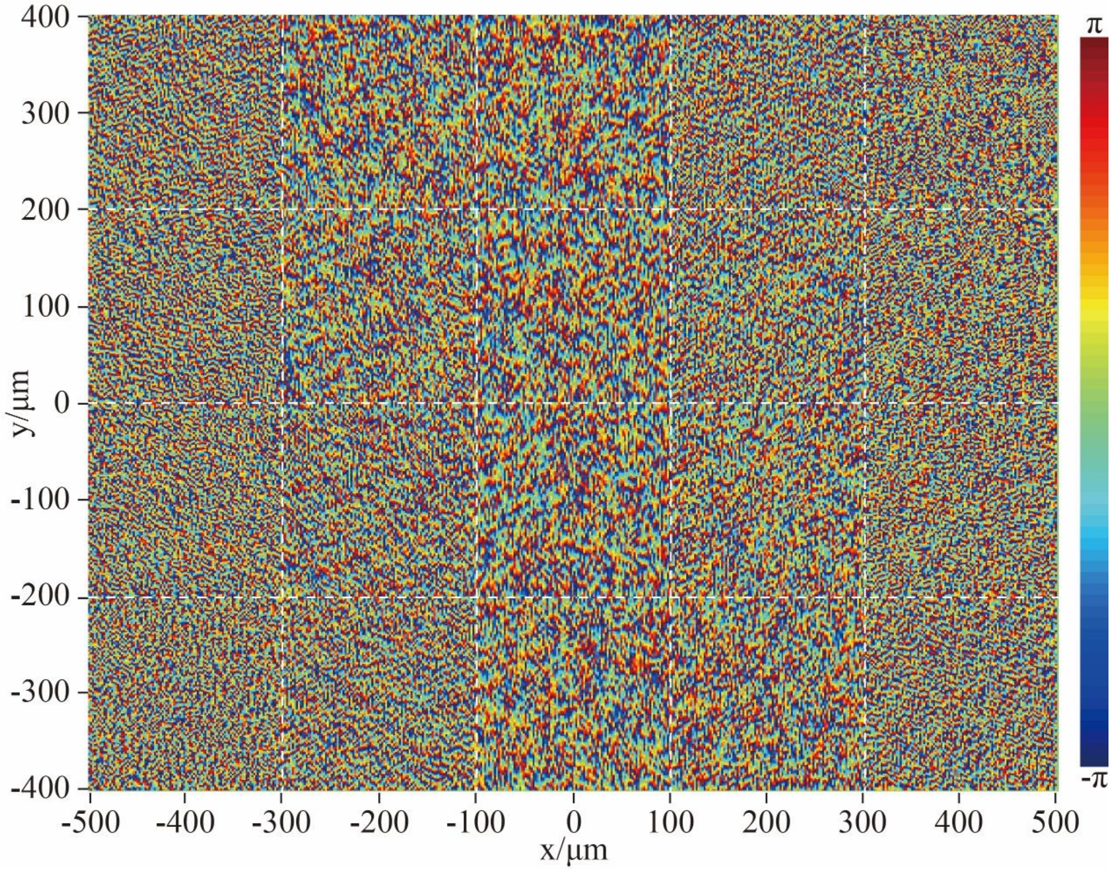

Fig. S3. Phase map of dynamic space-channel selective meta-hologram sample. The size of each space-channel is 200×200 μm^2 and the whole size of the fabricated metasurface region is 1000×800 μm^2. The holographic images are designed based on the Fraunhofer diffraction theory.

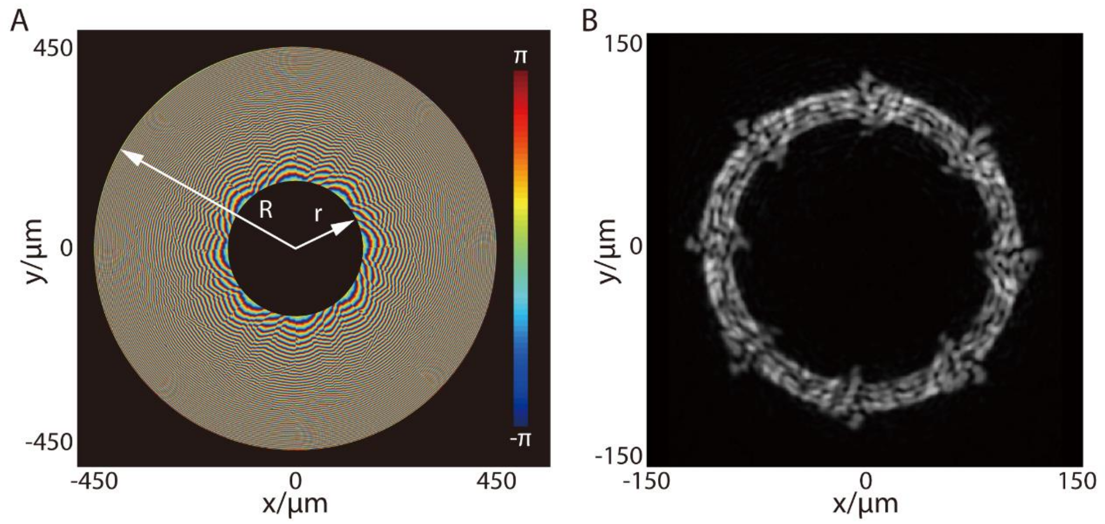

Fig. S4. Dynamic 3D space-channel selective meta-hologram. (A) Phase map of the 3D space-channel selective meta-hologram sample. Metasurface is divided into 8 space-channels, and each space-channel is designed to reconstruct a 3D arrow in free space. Geometrical parameters are marked in figure, and the internal radius of the annular metasurface is r = 150 μm while outer radius R = 450 μm. **(B)** The light field distribution of the reconstructed pattern at z = 2010 μm when all sub-regions are illuminated. The holographic objects are designed based on the Fresnel diffraction theory.

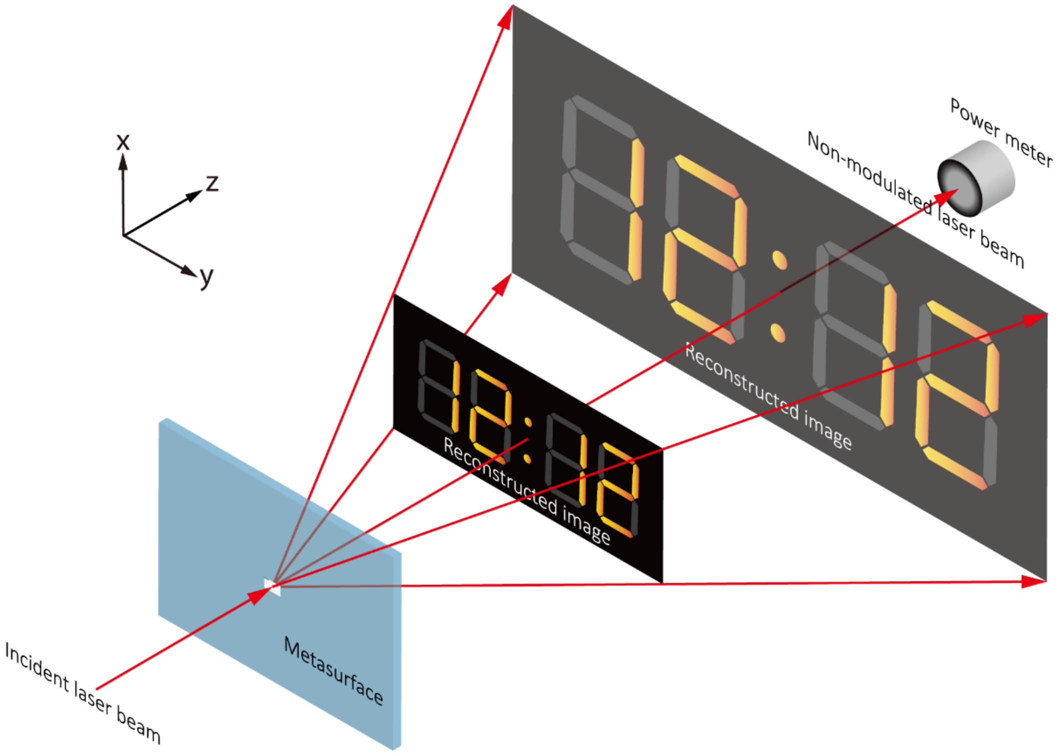

Fig. S5. The measurement of non-modulated laser beam.

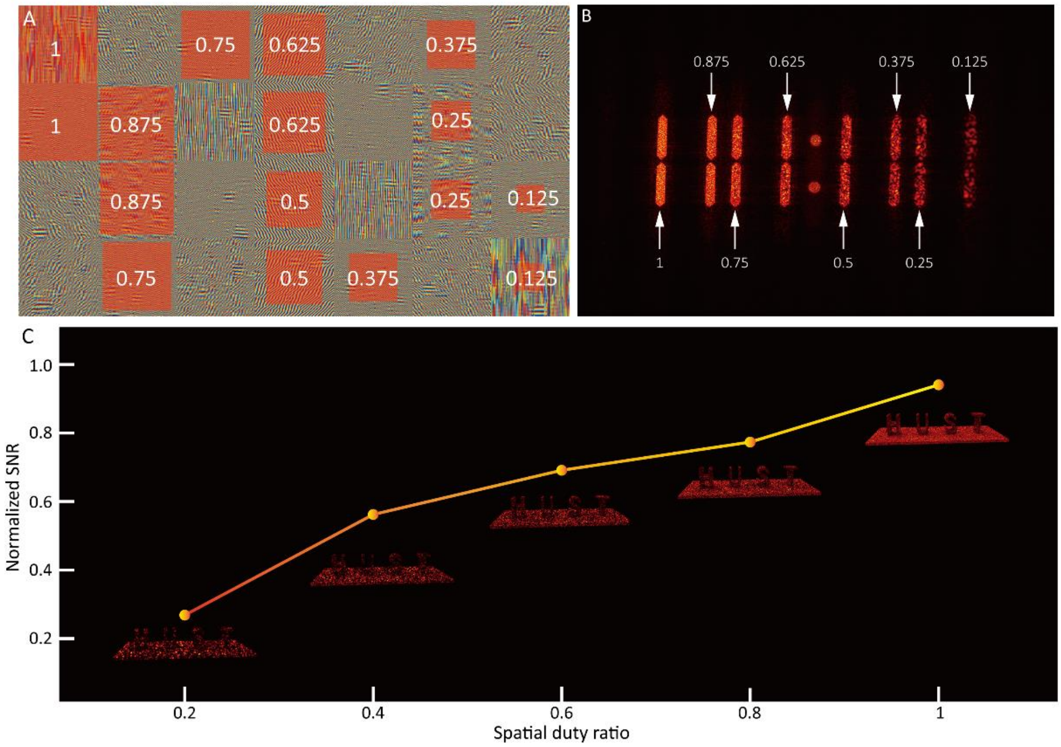

Fig. S6. Signal-to-noise analysis when space-channels are opened partly. The brightness and definition decay (**B**) when spatial duty ratio is reduced (**A**). The numbers marked in (A-B) is the spatial duty ratio of space-channels. **(C)** The signal-to-noise ratio (SNR) is defined as the ratio of the peak intensity in the image to the standard deviation of the background noise, which changes at different detection distance for samples designed by Fraunhofer diffraction. Thus, normalized SNR is presented in (C) to analyze the evolution of SNR to spatial duty ratio.

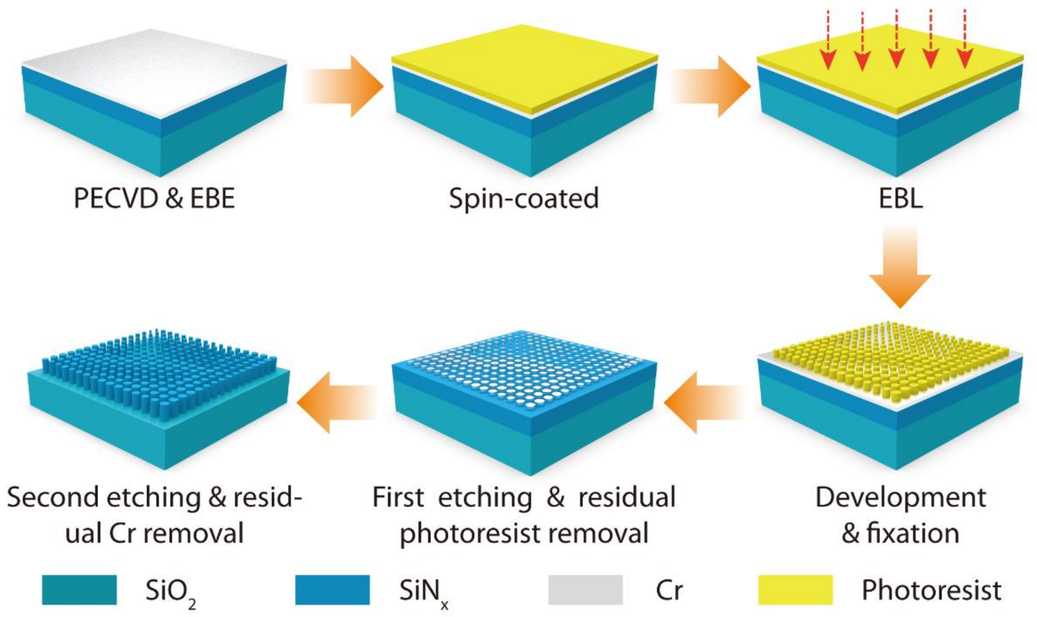

Fig. S7. Fabrication process of the SiN_x metasurface. The fabrication of the SiN_x metasurface starts from a glass wafer substrate with a thickness of 500 μm. A silicon nitride layer (n = 2.023 at 633 nm) of 700 nm thickness is deposited by plasma-enhanced chemical vapor deposition onto the substrate. Then, a chromium layer of 20 nm is deposited by electron-beam evaporation on top of the SiN_x layer as a hard mask. Next, a 200 nm photoresist layer (CSAR62) is spin-coated onto the top of the Cr layer. The hologram pattern is written by electron-beam lithography (EBL, Vistec: EBPG 5000 Plus) and implemented into the photoresist layer after development. The pattern is then transferred into the Cr hard mask layer by inductively coupled plasma etching (ICP, Oxford Plasmalab: System100-ICP-180), and the residual photoresist is stripped off by an oxygen plasma stripper (Diener electronic: PICO plasma stripper). Finally, the pattern is transferred into the SiN_x layer by the next ICP process, and the remaining Cr is removed by Cr corrosion solution. The Cr layer is utilized as a hard mask because of the extremely high etching selectivity between Cr and SiN_x.

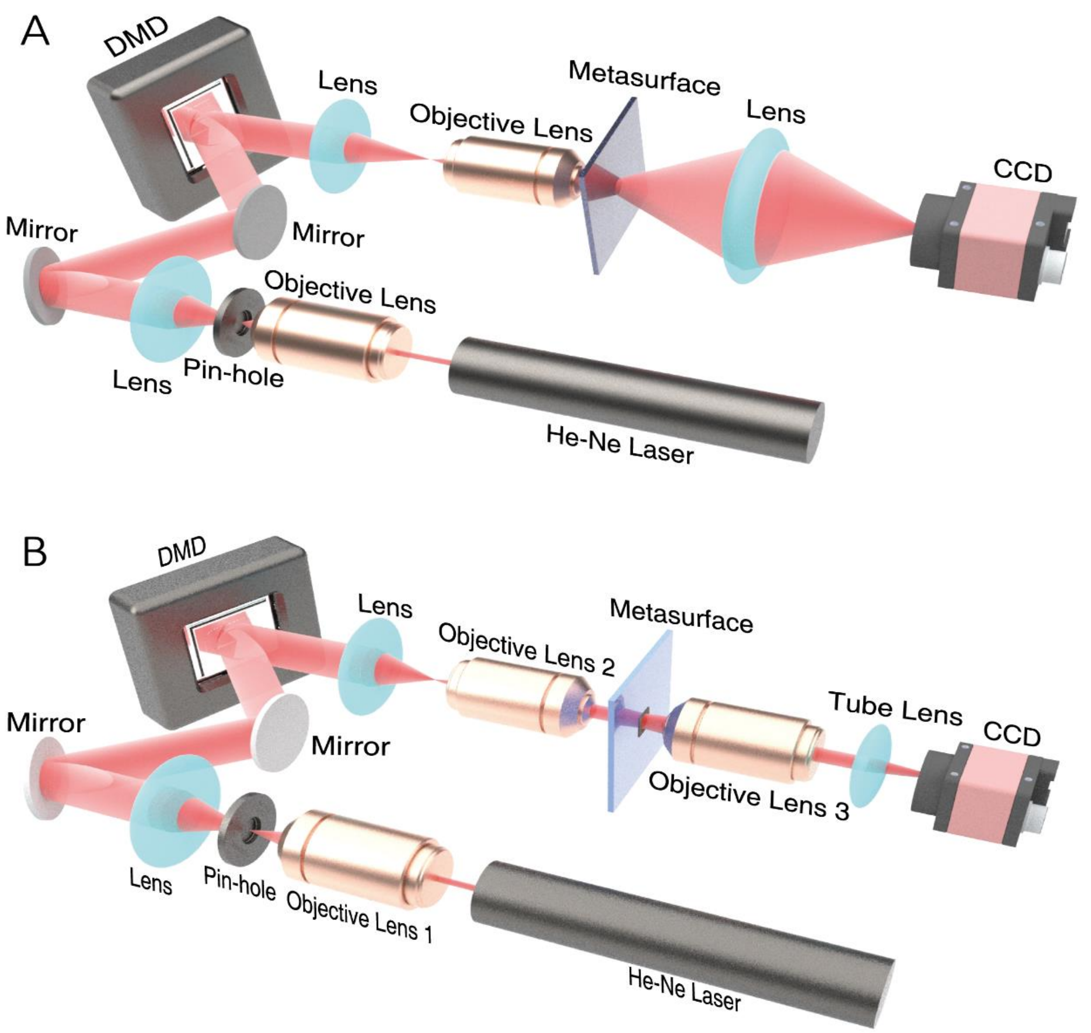

Fig. S8. Schematic of the optical setup. The optical components and setup of the dynamic space division multiplexing metasurface are shown in Fig. S8. The He-Ne laser (Pacific Lasertec, 25-LHP-991-230) at the wavelength of 633 nm propagates through a spatial pin-hole filter and collimating lens and becomes an expanded laser beam with suitable beam quality. Then, the expanded laser beam is modulated by a DMD (Texas Instruments, DLP6500FYE) at high speed. The coded beam propagates through the 4f system consisting of a lens and a microscope objective. The reconstructed holographic frames are collected by Fourier lens (**A**) or objective lens (**B**) and recorded by CCD.

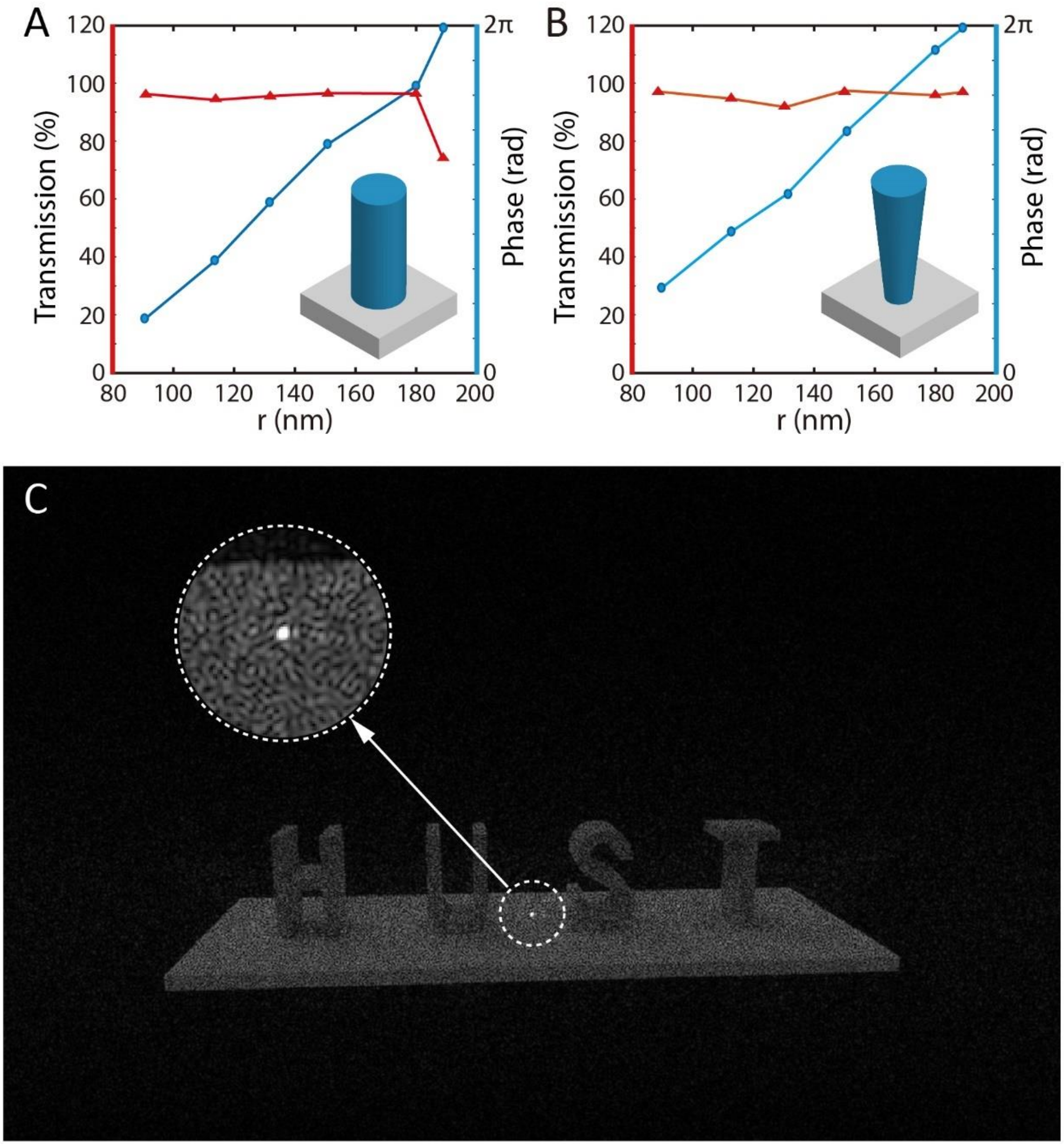

Fig. S9. Simulation results of tapering effects of nanopillars. (A) The amplitude transmission efficiency and phase response of SiN_x nanopillars with no fabrication errors. (B) The amplitude transmission efficiency and phase response of SiN_x nanopillars with tapered shape fabrication errors. (C) The simulated holographic reconstructed image with phase shift errors.

Movie S1. The display results of dynamic space-channel multiplexing meta-hologram. The left part is the space-channel coding pattern and enlarged structured laser beam while the right part is corresponding dynamic holographic patterns. The experimental result video has been adjusted a bit for better exhibition, such as zooming on the areas of holographic image and adjusting tilt angle.

Movie S2. The display results of dynamic space-channel selective meta-hologram. The left part is the scanning sequence while the right one is the experimental results. The experimental result video has been adjusted a bit for better exhibition, such as zooming on the areas of holographic image and adjusting tilt angle.

Movie S3. The display results of dynamic 3D space-channel selective meta-hologram. The movie shows the sketch map of design (3s~10s) and the experimental results (11s~14s). In the experimental results part, the right one is the light field distributions at z = 2010 μm and left one is the reconstructed 3D experimental results which are detected by the optical setup shown in fig. S8 (B). The experimental result video has been adjusted a bit for better exhibition, such as zooming on the areas of holographic image and adjusting tilt angle.
